# Lignin Derivatives in Chestnut Wood Hydrochar: Mild Solvolysis and Characterization

**DOI:** 10.1002/cplu.202400697

**Published:** 2025-03-28

**Authors:** Valeria Pierpaoli, Miha Grilc, Giorgio Tofani, Blaž Likozar, Edita Jasiukaitytė‐Grojzdek, Enrico Segoloni, Marco Barbanera, Manuela Romagnoli

**Affiliations:** ^1^ Department for Innovation in Biological, Agro-Food and Forest Systems (DIBAF), Institution Tuscia University via S. Camillo de Lellis, snc 01100 Viterbo Italy; ^2^ Department of Catalysis and Chemical Reaction Engineering, Institution National Institute of Chemistry Hajdrihova 19 1000 Ljubljana Slovenia; ^3^ Center of Excellence for Low Carbon Technology Hajdrihova 19 1001 Ljubljana Slovenia

**Keywords:** Hydrothermal treatment, Lignin, Tannin extraction, Exhausted wood

## Abstract

*Castanea sativa* plays a significant role in the Italian forestry sector, covering 7.5 % of Italy's land. This biomass resource includes significant volumes of wood processing residues: in recent years, its exploitation has shifted from fuel production to high‐value applications, focusing on lignocellulosic biomass fractionation to obtain biopolymers. Lignin, the most abundant aromatic biopolymer, has gained attention due to its potential for various industrial uses. Amid chemical treatments, hydrothermal treatment offers a sustainable approach to generate energy and chemicals simultaneously.

As depicted in Figure 1, the goal of this study is to evaluate the hydrochar from the hydrothermal treatment of chestnuts to determine the presence of lignin or resulting derivatives which can be isolated for high‐value applications. Virgin wood and detannized wood were processed in a hydrothermal reactor at 200 °C. The resulting hydrochar was characterized using FTIR and 2D NMR for functional group analysis, SEM for char morphology, HPSEC for molecular weight determination, TGA for thermal properties and the Folin Test to confirm the preservation of lignin structure post‐treatment. Preliminary results indicate lignin is present in certain fractions, suggesting the potentiality of this biomass for lignin production.

## Graphical abstract

This research investigates the effects of hydrothermal treatment on hydrochar derived from virgin wood and detannized wood, focusing on the preservation of lignin content. Results from Fourier Transformed Infrared Spectroscopy, solubility tests, scanning electron microscopy (SEM), Folin‐Ciocalteau analysis and High Performance Size Exclusion Chromatography (HPSEC) highlight significant structural changes and the potential for lignin valorization.

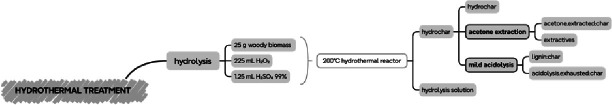

## Introduction

**Figure 1:**
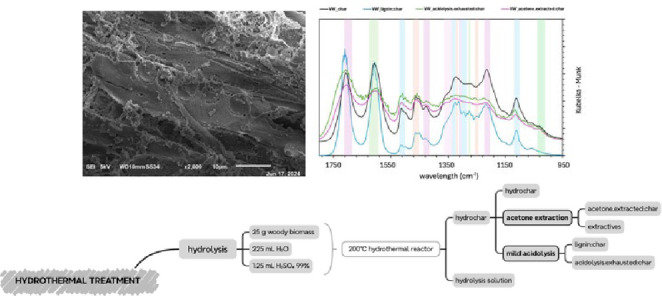
Graphical abstract.

Lignocellulosic biomass is an Earth‐abundant resource (about 200 billion tons/year) that holds significant potential for high‐value applications, making it a promising candidate for biomaterials even in biomedical and biotechnologies applications[^1^, ^2^, ^3^, ^4^, ^5^, ^6^] so as in chemical intermediates, which can replace fossil‐based resources. However, the potentiality of lignocellulosic residues for feedstock in biorefinery is currently only partially exploited.^[7]^ One of the most interesting feedstocks that can still be highly valorized is the waste from the chestnut wood processing industry. Chestnut (*Castanea sativa Mill.)* is a species with a fairly wide range in Europe and it is considered one of the species of the future, especially in light of climate change.^[8]^ The processing in sawmills of chestnut wood alone is estimated to produce a 40 % by volume of residues, which are chips or wood trimmings (Figure 1).

Another significant source of residue comes from the tannin extraction industry, which is present in Europe with three plants: two in Italy and one in Slovenia. Tannins extracted from chestnut wood are valuable compounds with applications across various sectors, including pharmaceutical, cosmetic, leather processing and animal feed.[^8^, ^9^, ^10^] Currently, lignocellulosic residues from the tannin industry, along with those from sawmills and other industries, are primarily managed through combustion for energy production.[^11^, ^12^, ^13^]

Lignocellulosic feedstocks contain three main biopolymers: cellulose (35–50 %), hemicellulose (20–35 %) and lignin (5–30 %). Indeed, even after post‐extraction, it has been shown that the exhausted biomass residues from the tannin industry still have interesting properties,[^12^, ^14^] and they could find alternative uses in value‐added supply chains.[^15^, ^16^, ^17^, ^18^] In particular, they could present promising resources for high‐value applications,^[13]^ contributing to circular economy. One potential avenue for valorizing lignocellulosic residues from chestnut wood supply chain consists of thermal treatments for biomass and, specifically, in the hydrothermal treatment (HTT). This process has interesting characteristics because it generates valuable compounds in the hydrolyzed liquid, such as xylo‐oligosaccharides, furfural, HMF and hydrochar, which have applications ranging from functional foods and pharmaceuticals to soil enhancement, bioplastics, carbon capture and sustainable fuel production.[^5^, ^14^, ^19^, ^20^, ^21^, ^22^, ^23^, ^24^, ^25^, ^26^] In recent years, there has been an increasing interest in valorizing diverse products from these thermochemical processes. For instance, xylo‐olygosaccharides can be isolated for use in health‐related products,[^20^, ^23^, ^27^] while furfural and HMF serve as precursors for various bio‐based chemicals,[^28^, ^29^, ^30^, ^31^] lignin also can be isolated from the hydrolysate.[^32^, ^33^]

Lignin has aroused special attention as it is the most abundant aromatic biopolymer on Earth. It is a complex polyphenolic macromolecule primarily composed of three basic monomeric units, p‐hydroxyphenil (H), guaiacyl (G) and syringyl (S) interconnected by β‐O‐4 linkages, among others.[^34^, ^35^, ^36^, ^37^, ^38^, ^39^] The proportion of both monomeric units and the type of linkages in lignin structure can vary according to the biomass source (softwoods, hardwoods or grasses), which affects lignin's mechanical and chemical properties.[^17^, ^40^]

The functional groups in lignin (methoxy, carbonyl, carboxyl and hydroxyl groups) are important active sites for further chemical modifications: synthesis of new chemically active sites, depolymerization or fragmentation, among others.[^17^, ^41^, ^42^, ^43^]

Indeed, the obtained modified lignin products can be used in several fields, such as phenolic resins, adhesives, bioplastics and even in the fabrication of nanoparticles for use as drug carriers.[^35^, ^44^, ^45^, ^46^, ^47^, ^48^, ^49^, ^50^, ^51^, ^52^, ^53^, ^54^] However, despite its potential, only 5 % of lignin is currently used for high‐value applications: its underutilization highlights the need to develop efficient lignin isolation processes.^[55]^ Hydrochar containing lignin or its derivatives owns desirable properties for some applications such as adsorptive removal of wastewater pollutants.^[56]^ From the chemical point of view, the structure of hydrochar is a carbonaceous cross‐linked polymer and has similar acid digestion properties as lignin, so the hydrochar is almost challenging to distinguish from unreacted lignin fraction without the use of specific techniques such as NMR.[^57^, ^58^] Thus, a linear polymer like cellulose can be converted into a cross‐linked polymer similar to lignin.^[59]^ Despite the significant advancements in the use of thermochemical products, there remains a notable gap in the literature regarding valorization of lignin trapped in char and full chemical knowledge. As far as we know, there is currently not fully established methodology for isolating lignin or its derivatives from char.^[60]^ In this research, the acidolysis process, a treatment already studied for lignin isolation from wood.^[61]^ This presents an exciting opportunity for future research to explore the potential of char‐derived lignin from waste chestnut, which remains an underexplored area within the broader field of biomass valorization.

The objective of our study is to utilize waste chestnut biomass as the starting material for this research. As a first step, it is essential to characterize the solid product (hydrochar) generated in the hydrothermal reactor to ensure that it contains lignin suitable for further applications. Following this characterization, we have attempted to isolate lignin or its derivatives using both acetone extraction and acidolysis techniques.

## Results and Discussion

**Scheme 1:**
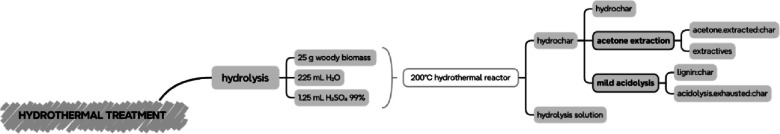
Hydrothermal treatment process scheme applied in this study to produce acetone extracted char and extractives (acetone extraction of hydrochar) and “lignin_char” (after mild hyrochar hydrolysis).

In this study the hydrothermal treatment process was explored as it is shown in Scheme 1. This treatment has been conducted twice: the first time on the virgin wood and the second time on the detannized wood. The main step of the treatment is the hydrolysis: 25 g of woody milled biomass were introduced into the hydrothermal reactor with 225 mL of H_2_O and 1.25 mL of H_2_SO_4_ 99 %. This reaction occurs in a high pressure reactor that was heated at a linear rate of 2.3 °C/min and held at 200 °C for 2 hours. After hydrolysis, two main fractions are generated: a liquid one, namely “hydrolysis solution” and a solid one, namely “hydrochar”. The hydrochar, on which this study is focused on, is subjected to further processing: i) an acetone extraction to produce an acetone extracted char and extractives, ii) a mild acidolysis which produces two fractions, namely “lignin_char” and “acidolysis.exhausted_char”. Mass balances for the process are shown in Table 1 and Table 2. All fractions were further characterized using FTIR, SEC and SEM.

**Table 1:** Mass balance of treatments applied to VW biomass, with yield referred to 25 g of biomass. Qi=initial quantity, Qo=obtained quantity. Yield based on the starting quantity.

Starting material	Process step	Obtained material	Qi (g)	Qo (g)	Yield (%)
VW	hydrothermal treatment	VW_hydrochar	25.00	9.72	38.88
VW_hydrochar	acetone extraction	VW_acetone. extracted : char	4.86	4.37	17.52
VW_hydrochar	mild acidolysis	VW_acidolysis. exhausted : char	4.86	0.92	3.69

**Table 2:** Mass balance of treatments applied to DT biomass, with yield referred to 25 g of biomass. Qi=initial quantity, Qo=obtained quantity. Yield based on the starting quantity.

Starting material	Process step	Obtained material	Qi (g)	Qo (g)	Yield (%)
DT	hydrothermal treatment	DT_hydrochar	25.00	9.71	38.84
DT_hydrochar	acetone extraction	DT_acetone. extracted : char	4.85	3.92	18.40
DT_hydrochar	mild acidolysis	DT_acidolysis. Exhausted : char	4.85	1.60	6.41

### Fourier Transformed Infrared Spectroscopy

**Figure 2:**
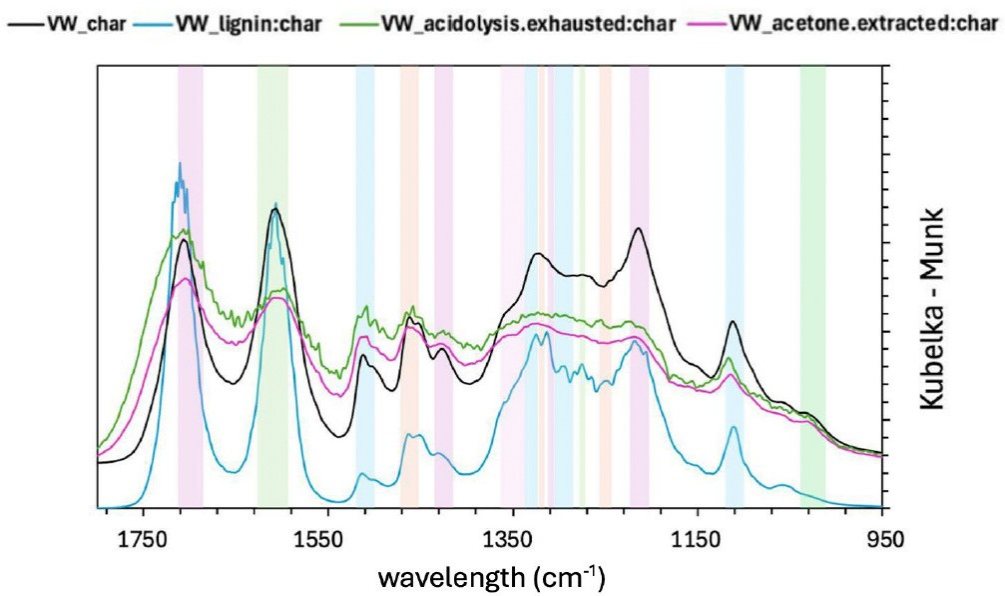
FTIR of VW char right after the hydrothermal treatment (VW_char), after the acidolysis treatment (V?_lignin : char) and also after the acetone extraction (VW_acetone.extracted:char) showing some typical lignin peaks. On the other hand, the exhausted char after the acidolysis treatment does not have the same typical lignin peaks and happens to show a slightly different profile (VW_acidolysis.exhausted:char).

**Figure 3:**
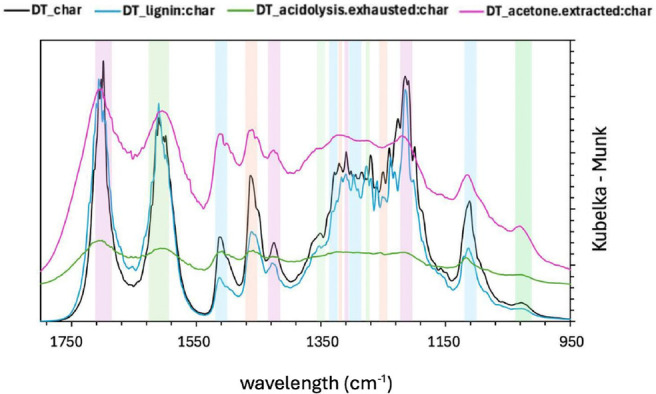
FTIR of DT char right after the hydrothermal treatment (DT_char), after the acidolysis treatment (DT_lignin : char) and also after the acetone extraction (DT_acetone.extracted:char) showing some typical lignin peaks. On the other hand, the exhausted char after the acidolysis treatment does not have the same typical lignin peaks and happens to show a slightly different profile (DT_acidolysis.exhausted:char).

The FTIR spectra of virgin wood char (VW) and detannized wood char (DT) in the different steps of the hydrothermal treatment are shown in Figure 2 and Figure 3. The peak assignments were conducted according to the literature.[^62^, ^63^, ^64^, ^65^] and are summarized in Table 3 and 4.

**Table 3:** Main functional groups and peaks of the samples. A: VW_char, B: VW_lignin : char, C: VW_acetone.extracted : char, D: VW_acidolysis.exhausted:char.

Wavenumber (cm^−1^)			Base group and vibration mode	
A	B	C	D	
1715	1711	1710	1710	C=O stretch in unconjugated ketone, carbonyl and in ester groups (frequently of carbohydrate origin)^[65]^
1612	1611	1600	1605	C=O stretching conjugated to aromatic ring^[64]^
1510	1510	1510	1510	aromatic skeletal vibration plus C=O stretching[^64^, ^65^]
1460	1462	1464	1467	C−H deformation stretching in lignin and carbohydrates, C−H deformation (methyl and methylene)[^64^, ^65^]
1418	1425	1427	1426	aromatic skeletal combined with C−H in‐plane deforming and stretching^[65]^
1326	1319			condensation of G unit and S unit, S unit and CH_2_ bending stretching[^64^, ^65^]
	1309			condensation of G and S units, S unit and CH_2_ bending stretching, O−H bending^[64]^
	1275			guaiacyl ring breathing plus C=O stretching[^64^, ^65^]
	1250			C−O lignin and mannosan^[63]^
1216	1211			C−O of G ring^[62]^
1115	1110	1105	1120	aromatic skeletal and C−O stretch^[65]^
1029	1053	1027	1022	C−O deformation in primary alcohols, plus C=O stretch unconj., plus aromatic C−H in‐plane deformation^[65]^

**Table 4:** Main functional groups and peaks of the samples. E: DT_char, F: DT_lignin : char, E: DT_acetone.extracted:char, G DT_acidolysis.exhausted:char.

Wavenumber (cm^−1^)			Base group and vibration mode	
E	F	G	H	
1700	1711	1708	1710	C=O stretch in unconjugated ketone, carbonyl and in ester groups (frequently of carbohydrate origin)^[65]^
1616	1610	1608	1605	C=O stretching conjugated to aromatic ring^[64]^
1510	1510	1513	1510	aromatic skeletal vibration plus C=O stretching[^64^, ^65^]
1462	1462	1458	1467	C−H deformation stretching in lignin and carbohydrates, C−H deformation (methyl and methylene)[^64^, ^65^]
1425	1425	1429	1426	aromatic skeletal combined with C−H in‐plane deforming and stretching^[65]^
1319	1319			condensation of G unit and S unit, S unit and CH_2_ bending stretching[^64^, ^65^]
1309	1309			condensation of G and S units, S unit and CH_2_ bending stretching, O−H bending^[64]^
1275	1275			guaiacyl ring breathing plus C=O stretching[^64^, ^65^]
1250	1250			C−O lignin and mannosan^[63]^
1220	1213			C−O of G ring^[62]^
1110	1110	1120	1120	aromatic skeletal and C−O stretch^[65]^
1025	1025	1027	1025	C−O deformation in primary alcohols, plus C=O stretch unconj., plus aromatic C−H in‐plane deformation^[65]^

The fingerprint region of the FTIR spectrum is characterized by a high degree of complexity, attributed to the presence of numerous bands from the primary components of wood. These bands result from various functional groups within the wood's constituents. Notably, distinct bands around 1599, 1510, 1270 and 1240 cm^−1^ are linked to the specific bending or stretching of groups associated with lignin. Similarly, bands at 1463, 1423, 1110 and 1030 cm^−1^ are related to characteristic vibrations found in both lignin and cellulose.^[65]^ In all the samples for both VW and DT, before and after extraction with acetone and after the mild acidolysis, major peaks, such as those corresponding to the stretching vibrations of C=O in aromatic compounds and C−O, typically appear around 1510 and 1030 cm^−1^.^[64]^ Vibrational modes like O−H, C−H and C=O above 1600 cm^−1^, along with the aromatic skeletal vibrations near 1510 cm^−1^ are notably distinct while the 1600 cm^−1^ band is broadened, especially after the acetone extraction and the mild acidolysis, likely due not only to the overlap with C=O stretching^[64]^ but also due to a reduced structural integrity following acetone extraction and mild acidolysis treatments, which lead to lignin degradation. This effect is even more pronounced in the DT sample compared to the un‐extracted one, as the tannins in the un‐extracted material contribute to the aromatic bands, given their aromatic structure. Furthermore, below 1460 cm^−1^ a more intricate pattern emerges, primarily due to contributions from carbohydrate and lignin vibrations.^[62]^

It should be emphasized that the majority of the bands in this region reflect input from all wood components, making the interpretation of IR data challenging. However, shifts in peak positions and variations in intensity are evident when comparing charred wood in the various treatment steps.

All the samples exhibit a signal at 1710 cm^−1^ to be attributed to C=O vibrations corresponding to carbonyl, ester or carboxyl.^[66]^

The spectra of the fingerprint region of the four char samples of the virgin wood are rather similar as 7 out of the 13 discernible bands are shared by all the samples. The lignin‐containing char (VW_lignin : char) exhibits more bands compared to the other virgin wood char samples in the region between 1319 and 1250 cm^−1^. The VW_char sample appears to be the more similar to the VW_lignin : char as it shares 8 out of the 12 discernible bands with it.

Similar conclusion can be drawn for the detannized char samples: the samples share 7 out of 12 discernible bands and the DT_char and DT_lignin : char are the most similar samples, sharing 11 bands.

Having a look at the VW_char and VW_lignin : char after the mild acidolysis, it can be noted that new bands occurred such as the ones at 1309, 1303, 1275, 1250 cm^−1^: all these bands are attributed to the lignin constituent of wood. Also, among the differences between VW_char and DT_char, the bands at 1309, 1275 and 1250 cm^−1^, which are associated with lignin, are visible in the DT_char but not in VW_char. The chestnut is rich in tannins, which certainly contribute to the lignin peaks since it is a phenolic and aromatic compound – that can be seen in the VW samples, unlike in the DT samples, since most of the tannins had been removed. This suggests that detannization may emphasize lignin that would otherwise be less accessible. Thereby, the presence of phenols and tannins in VW_char may interfere with the visibility of bands associated with lignin structures, making it more challenging to detect lignin in the VW_char.

The spectra of DT_char and DT_lignin correspond mostly because the tannin portion has been removed, unlike the VW samples. Having a look at the detannized wood char before and after the mild acidolysis, the onset of the band at 1303 cm^−1^ can be observed. Other bands disappear when treating the char with the acetone extraction and on the exhausted char obtained through the acidolysis such as the ones at 1319, 1309, 1275, 1250, 1213 cm^−1^, which are lignin‐related. Thereby, a common feature in both cases is the flattening of the spectrum after acidolysis in the acidolysis exhausted hydrochar samples – and also after acetone extraction, though to a lesser extent.

In the end, the comparison between char samples before and after acidolysis – for both virgin wood and detannized wood – reveals the appearance of new bands which are attributed to lignin. This suggests that the treatment causes a breaking of covalent bonds of wood structure, allowing the release or transformation of lignin, making new vibrations in the aromatic structures detectable. Moreover, the absence of certain bands after acetone extraction and after acidolysis in the exhausted sample confirms that the treatment removes specific lignin components.

To draw some conclusions, it can be said that the changes observed in the FTIR spectra indicate that the acidolysis affects the lignin content in the wood also after it has been hydrothermally‐treated and that a lignin enriched fraction can be obtained.

### Scanning Electron Microscopy (SEM)

**Figure 4:**
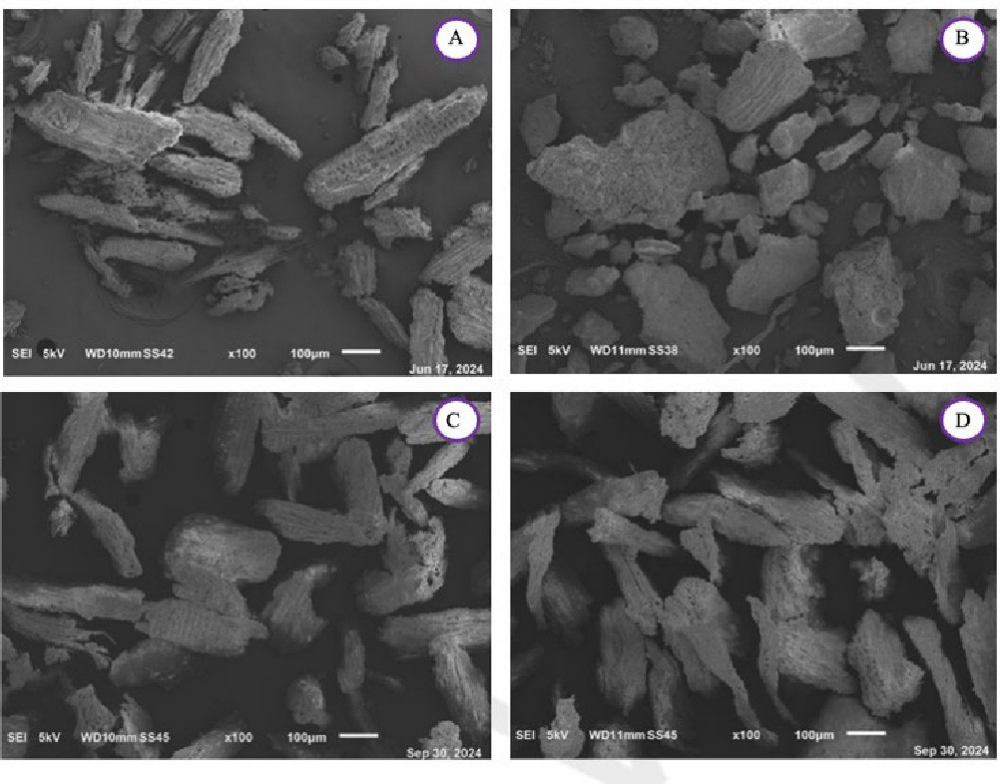
SEM images at a magnification of ×100 of A: virgin char after the hydrothermal treatment (VW_char), B: virgin char after the acidolysis treatment (VW_lignin : char), C: virgin char after the acetone extraction (VW_acetone.extracted:char), D: virgin exhausted char after the acidolysis treatment (VW_acidolysis.exhausted:char).

**Figure 5:**
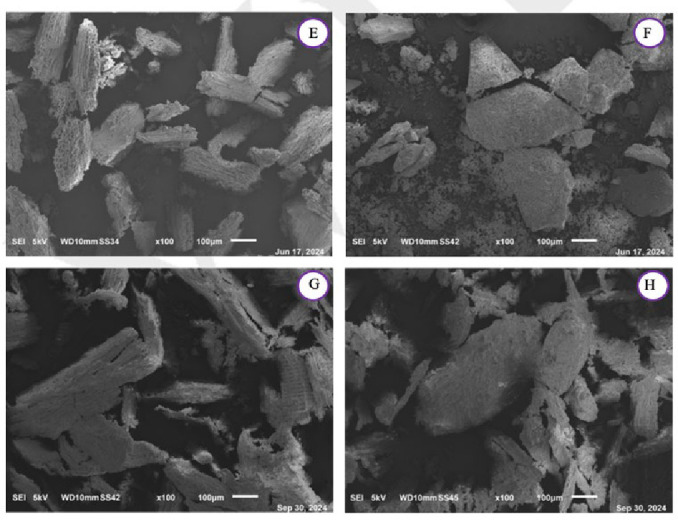
SEM images at a magnification of ×100 of E: detannized char after the hydrothermal treatment (DT_char), F: dettanized char after the acidolysis treatment (DT_lignin : char), G: detannized char after the acetone extraction (DT_acetone.extracted:char), H: detannized exhausted char after the acidolysis treatment (DT_acidolysis.exhausted:char).

**Figure 6:**
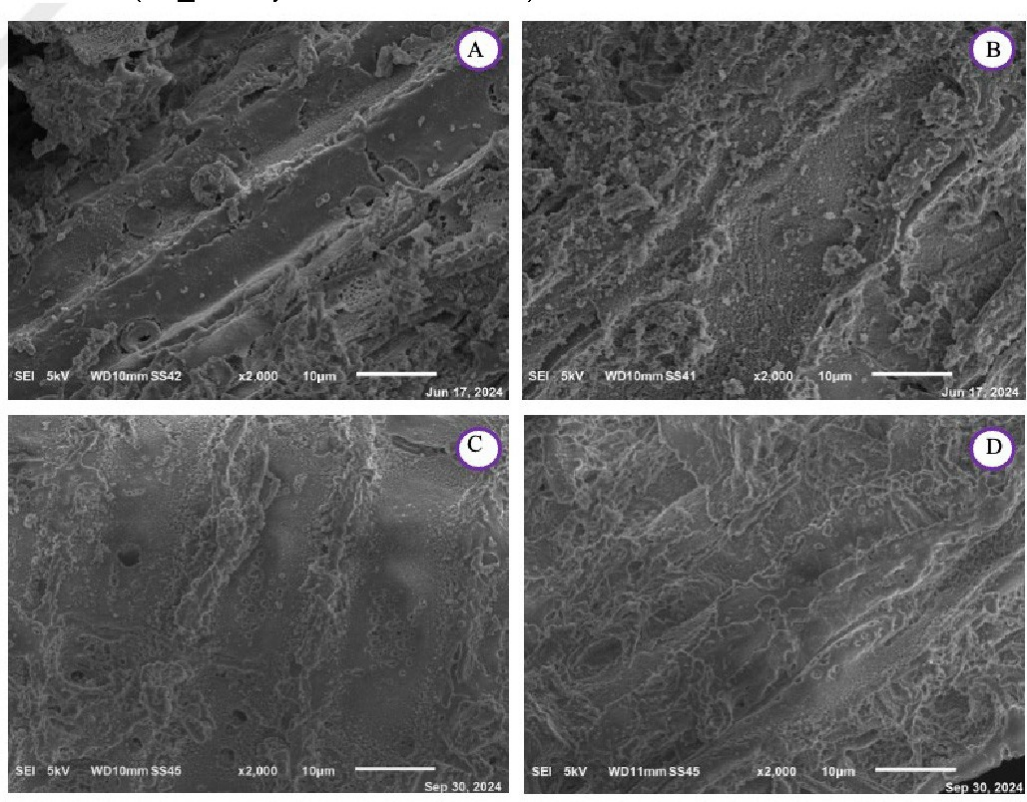
SEM images at a magnification of ×2000 of A: virgin char after the hydrothermal treatment (VW_char), B: virgin char after the acidolysis treatment (VW_lignin : char), C: virgin char after the acetone extraction (VW_acetone.extracted:char), D: virgin exhausted char after the acidolysis treatment (VW_acidolysis.exhausted:char).

**Figure 7:**
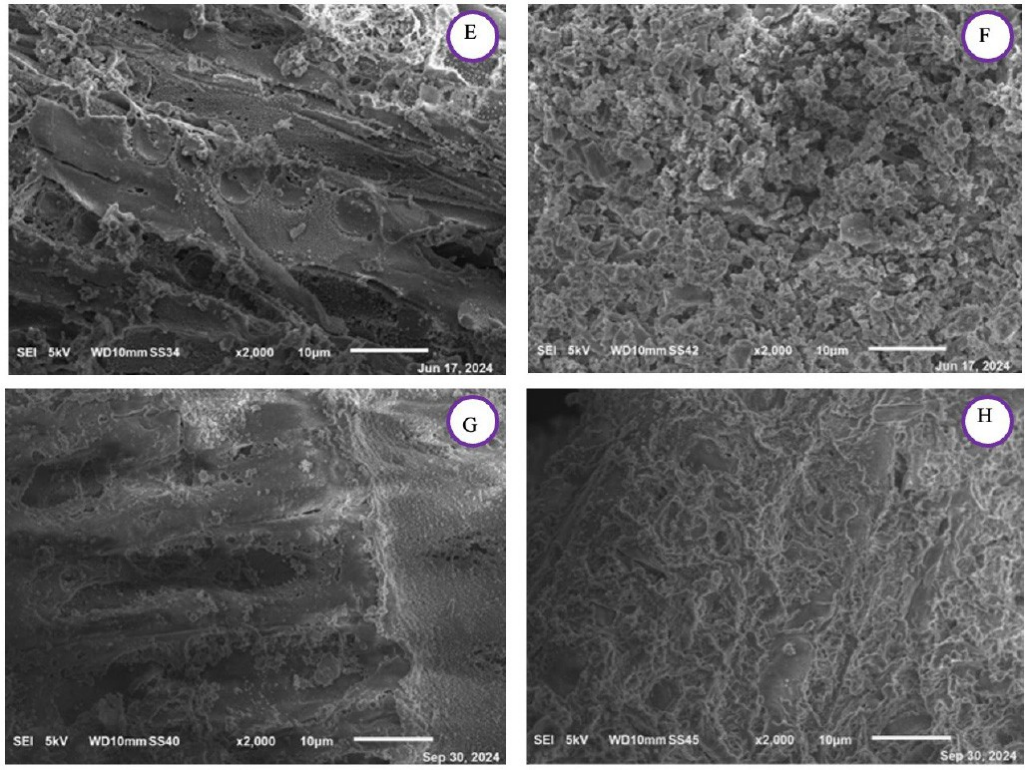
SEM images at a magnification of ×2000 of E: detannized char after the hydrothermal treatment (DT_char), F: dettanized char after the acidolysis treatment (DT_lignin : char), G: detannized char after the acetone extraction (DT_acetone.extracted:char), H: detannized exhausted char after the acidolysis treatment (DT_acidolysis.exhausted:char).

SEM imaging was performed on all the char samples and is shown at diverse magnification for the VW in Figure 4 and Figure 6 and for the DT in Figure 5 and Figure 7. As previously discussed, changes in the spectra of the respective samples are observed, attributed to the release of lignin within the samples after the mild acidolysis. In addition to this, a different particle aggregation is also noticeable. Notably, both the VW and the DT exhibit greater fragmentation following hydrothermal treatment compared to acidolysis, with VW consistently more fragmented than DT across all samples. A comparison between VW_char and DT_char reveals that in DT_char, the wood structure is less distinguishable, suggesting that detanizzation may already begin to compromise the integrity of the wood. Acetone extraction also degrades the cell wall, though to a lesser extent than acidolysis, which has already been noticed through the FTIR spectra.

After the acidolysis treatment, in the DT_lignin : char after the hydrothermal treatment, the original wood structure is still visible. Following acidolysis, the residue shows destruction of cell walls, resulting in a morphology more similar to wood powder, where individual cell elements are present. In the virgin wood char after the hydrothermal treatment, wood structure is visible, but after lignin extraction, the wood cells are less discernible due to a powderier appearance and increased particle aggregation. Moreover, increasing the magnification enables better observation of the sample textures, allowing for a more accurate comparison. The VW char obtained after the hydrothermal treatment displays a less jagged texture compared to that resulting from acidolysis: this is also evident in images c and d in Figure 6, where the surface of the particles in image d is less polished than image c.

In the end, it can be said that the mild acidolysis process leads to a reduction in the material's porosity, resulting in a visibly smoother surface compared to the initial condition.

### Folin‐Ciocalteu Analysis

The Folin‐Ciocalteu assay is typically used to measure phenol content, but since it reacts with all oxidizable groups, it primarily reflects reduction capacity that is linked to both the phenolic content and antioxidant activity.^[67]^ The aim was to determine whether the peaks observed in the FTIR spectrum, attributed to phenols in the char, were due to presence of free phenols – indicating that the hydrothermal treatment may have affected the lignin structure, causing depolymerization and degradation reactions, which is of key interest and should be preserved as much as possible – or if they were a result of the phenolic functional groups present in the lignin. The results in Table 5 show that the percentage of free phenols is extremely low (always below 0.06 wt %), indicating that the hydrothermal treatment did not compromise the key aspect of interest for further valorization, which is the lignin structure. Since the hydrothermal treatment may have led to the formation of C−C bonds, resulting in the loss of functional groups that were originally conducive to free radical oxidation, this can explain why the FC method yields such values for the phenolic content.

**Table 5:** Results obtained via Folin Test concerning the phenolic content.

Sample	Phenols content (wt %)
VW_char	0.03
VW_lignin : char	0.05
VW_acetone.extracted:char	0.05
VW_acidolysis.exhausted:char	0.05
DT_char	0.03
DT_lignin : char	0.06
DT_acetone.extracted:char	0.04
DT_acidolysis.exhausted:char	0.05

### High Performance Size Exclusion Chromatography (HPSEC)

**Figure 8:**
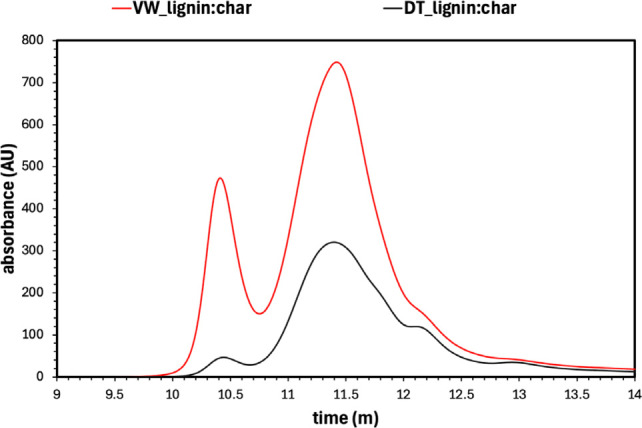
HPSEC chromatograms profiles of the VW_lignin : char (*red)* and DT_lignin : char (*black*) obtained via mild acidolysis. These profiles show that the VW_lignin : char sample has a greater abundance of high‐molecular weights species compared to the DT_lignin : char since it eluates first.

While confirming that the lignin structure similarities were identified, it is not sufficient on its own for the identification of lignin. Another crucial parameter is the molecular weight of the lignin. As an example, when lignin's molecular weight is low (between 500 and 3000 Da)^[68]^ it can be oxidized to produce vanillin or vanillic acid, which are both used as flavoring agents or to produce BTX, currently produced from petroleum, and which are source of 60 % of all aromatic volume. Moreover, it can be incorporated into polyurethane foams.^[69]^ Instead, to produce useful nanoparticles, the molecular weight in kDa must be as high as possible. For this reason, HSPEC analysis was also conducted on the lignin‐extracted fraction from the char, namely VW_lignin : char and DT_lignin : char and the chromatograms are shown in Figure 8 while the results are shown in Table 6. The data show that for both “lignin : char” fractions, the molecular weight is above 3 kDa. Moreover, the detannisation process allow to obtain a “lignin : char” fraction of lower molecular weight that starting from the virgin biomass. This behaviour is reasonable considering that the removal of tannins would allow for a easier breakdown of lignin's structure. It is important to note that the samples contain two peaks that allow us to consider the presence of lignin derivatives of different molecular weights, which also contribute to the observed chromatographic profiles. The dispersity (Đ) value is similar between the two fractions and broad (>4) meaning of heterogeneous mixtures of lignin derivatives fractions having various molecular weights. It is reasonable looking at the different peaks observed in Figure 8. In the case of the VW_lignin : char sample two main peaks are observed at elution time of around 10.5 and 11.5 min, respectively. Less prominent is a peak at around 12.25 min. It is interesting, observing that the DT_lignin : char sample still shows a peak at 10.5 min, but with significant lower intensity meaning that this sample contains less high molecular lignin derivatives fractions. At the same time, the peak at around 12.25 min becomes more evident. It is possible to suppose that the detannized sample underwent a major depolymerisation than the virgin one.

**Table 6:** Results obtained via HPSEC concerning the molecular weight.

Sample	M_n_ (kDa)	M_w_ (kDa)	Đ
VW_lignin : char	7.5	33	4.4
DT_lignin : char	3.5	15	4.3

### Solubility Tests

To evaluate the absence of polysaccharides in each sample, solubility tests were also performed using dimethyl sulfoxide (DMSO). The solubility of the samples in DMSO allowed us to infer the absence of polysaccharides which are insoluble in this solvent.^[70]^ The samples were visually inspected after 24 hours and just the VW_lignin : char and DT_lignin : char samples were found to be completely soluble.

### 2D HSQC NMR

The presence of two peaks at different retention times in the HPSEC chromatograms suggests that the sample consist of a mixture of lignin‐like fragments with different molecular weight. 2D HSQC NMR analysis of these samples was conducted, which confirms the presence of lignin and other derivatives, likely due to the extensive depolymerization/recondensation of phenolic materials in the original sample caused by the harsh reaction conditions.

**Figure 9:**
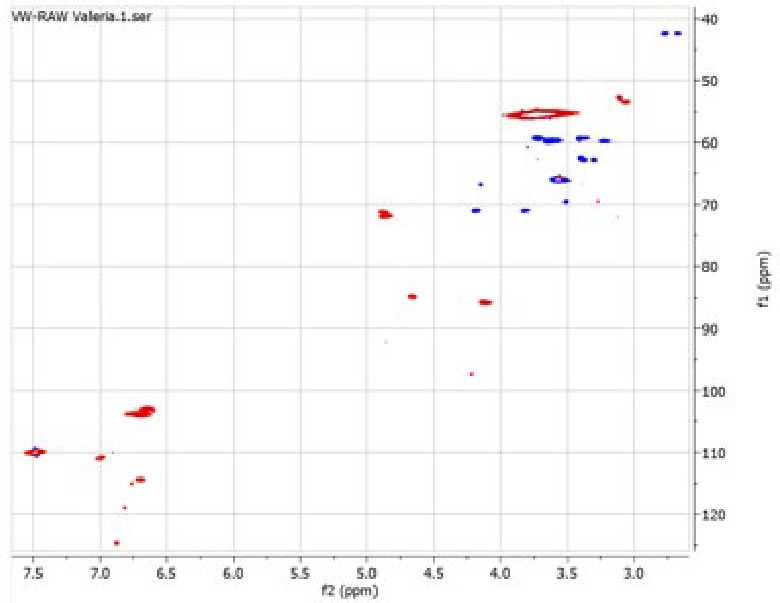
2D HSQC NMR of the “VW_lignin : char” (left) and of the “DT_lignin : char” (right).

**Figure 10:**
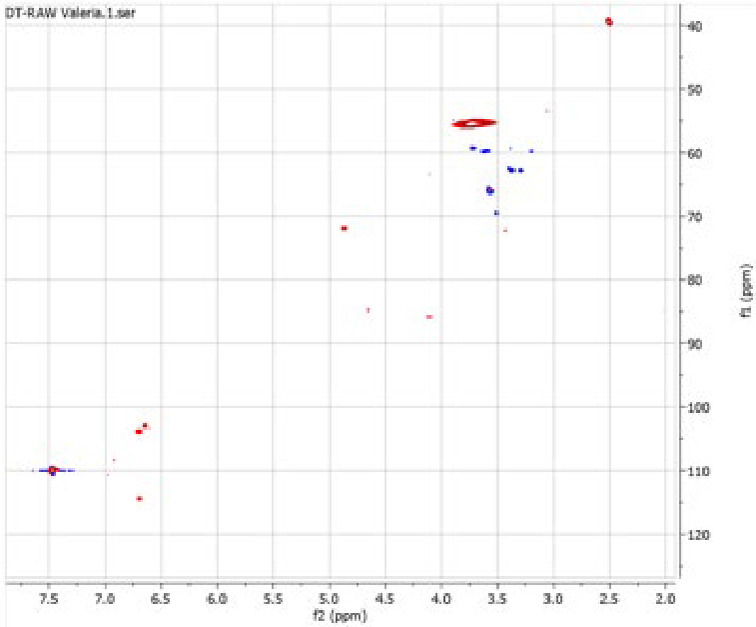
2D HSQC NMR of the “VW_lignin : char” (left) and of the “DT_lignin : char” (right).

For the virgin sample (Figure 9), the aromatic region exhibits correlation between 6.5 and 7.5 ppm (H) within 100 and 120 ppm (C). In the aliphatic region, several signals are observed between 3.25 and 5.0 ppm (H) and between 40 and 90 ppm (C). In the detannified sample (Figure 10), the aromatic region presents similar correlation as observed in the virgin sample. However, this sample displays a greater number of signals, in agreement with the FTIR analysis, which indicates that the removal of tannins enhances the detection of other aromatic compounds in the spectrum. Notably, signals associated with phenolic compounds are absent both in the virgin sample and in the detannified sample – with aromatic protons typically observed between 4.0 and 7.0 ppm and the aromatic carbons between 100 and 155 ppm. This absence of signals corresponding to aromatic in the NMR spectra further supports the findings from the Folin‐Ciocalteu assay, which showed a phenol content close to zero in both samples. Moreover, the NMR spectra show multiple β‐O‐4 linkages, with characteristic resonances corresponding to the different positions within the linkage: β‐O‐4_γ_ between 3 and 4 ppm (H) and between 60 and 65 ppm (C) as well as at 4.5 ppm (H) and 65 ppm (C); β‐O‐4_β_ between 4 and 5 ppm (H) and between 80 and 85 ppm (C); β‐O‐4_α_ at around 5 ppm (H) and 70 ppm (C). The presence of these signals strongly supports the presence of lignin in the sample, as the β‐O‐4 bond is the most abundant interunit linkage in lignin. While these types of bonds are also found in polysaccharides, the presence of a broad cross signal between δ_C_ 50–60 ppm and between δ_H_ 3.5–4.0 ppm, corresponding to methoxyl groups, suggests that these cross signals arise mostly from lignin. Furthermore, the absence of significant signals in the 90–110 ppm (C) aliphatic region also supports this interpretation. Additionally, signals corresponding to G units are observed at 7 ppm (H) and 110 ppm (C), while signals associated with S units appear between 6.5 and 7.0 ppm (H) and between 100 and 110 ppm (C), also confirming the presence of lignin.

The main conclusion is that the aromaticity characteristic to lignin is prominent, which accordingly confirms the presence of lignin in both samples. This observation confirms that the samples comprise a mixture of lignin‐like components – a finding that is also supported by the HPSEC analysis.

The assignment of the signals in the NMR spectrum was carried out based on literature data.[^71^, ^72^, ^73^, ^74^, ^75^, ^76^, ^77^]

### Thermogravimetric Analysis (TGA)

**Figure 11:**
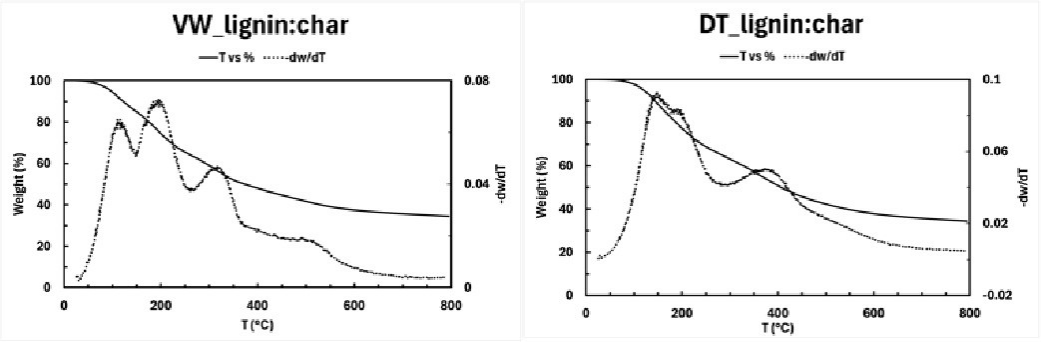
TGA of the “VW_lignin : char” (left) and of the “DT_lignin : char” (right).

The TGA graphs from which the data were obtained are displayed in Figure 11. As shown in Table 7, the “VW_lignin : char” sample has a lower T5 % which can be attributed to the presence of tannins in the sample: those compounds likely make the material more susceptible to degradation at lower temperatures. In contrast, “DT_lignin : char”, having undergone a process that removed them, shows a higher value, indicating a more thermally stable structure.

**Table 7:** Results concerning the TGA of the “VW_lignin : char” and the “DT_lignin : char”. T5 %is the temperature (°C) at which 5 % of the material's initial weight has been lost. %w 800 °C _N2_ indicates the percentage of the material's weight remaining at 800 °C, %ashes 800 °C _O2_ indicates the residue left after complete thermal decomposition.

Sample	T5 %	%w 800 °C N2	% ashes 800 °C _O2_
VW_lignin : char	97	35	11
DT_lignin : char	121	34	9

Moreover, the %w at 800 °C in N_2_ provides insights into the decomposition stages and residues and reflects the proportion of the material that does not decompose under inert conditions. The % ashes at 800 °C in O_2_ represents inorganic or non volatile components in the material. As shown in Table 7, the values for both parameters are comparable indicating that both samples have a comparable degree of thermal stability and share a similar composition in inorganic or non‐volatile components.

Accordingly to that conclusion, the T onset values under nitrogen atmosphere, as shown in Table 8, align with that results, showing how the thermal degradation process begins at a lower temperature for the “VW_lignin : char” sample compared to the “DT_lignin : char” sample. Additionally, it can be observed that three T_peak_ values and consequently three T_onset_ values are identified for both samples. T_peak_ represents the temperature at which the degradation reaction reaches its maximum rate for each decomposition step, while T_onset_ stands for the temperature at which significant thermal degradation begins. The presence of three distinct values aligns with the NMR and HP‐SEC results which suggest the presence of a mixture of derivatives decomposing at different temperatures.

**Table 8:** Results concerning the TGA of the “VW_lignin : char” and the “DT_lignin : char”. All the T values are in °C.

	VW_lignin : char	DT_lignin : char
T _N2peak;1_	115	150
T _N2onset;1_	88	110
T _N2peak;2_	197	195
T _N2onset;2_	161	181
T _N2peak;3_	318	380
T _N2onset;3_	276	330

## Conclusions

The research so far has demonstrated that after the hydrothermal treatment, the biochar exiting the reactor contains lignin. The Folin Test showed that the typical phenol peaks in the FTIR are not due to the presence of free phenols, but are related to the structure of lignin. Enriched lignin fraction can be separated from the cellulosic part of the char through purification treatments such as mild acidolysis. SEM analysis shows that the acidolysis treatment alters the biomass structure, resulting in increased aggregation and reduced porosity. HP‐SEC analysis reveals two chromatographic peaks for each sample, suggesting the presence of a mixture of lignin derivatives with different molecular weights. Solubility tests exclude the presence of polysaccharides, a finding supported also by H NMR, which indicates a significant presence of lignin‐derived structures. Additionally, TGA, used to study the thermal behaviour and stability of the material, shows three distinct thermal degradation steps for each sample, also confirming that the material is a mixture of lignin derivatives. Subtle differences observed in TGA and NMR spectra between the two samples are primarily attributed to the absence of tannins in the detannified sample.

The final product of this process is a char rich in lignin, as shown by the FTIR spectrum with a high molecular weight as shown by the HP‐SEC analysis. For this reason, this fraction shows the possibility to be further studied for the isolation of lignin to evaluate valorization processes.

## Materials and Methods

### Materials

The wood materials, virgin wood waste (VW) and detannified wood waste (DT) were provided by the Nuova Rivart (Srl), a tannins industry in Radicofani (SI, Italy). The VW has an intact state prior to any alterations or treatments, while the DT stands for the residual material after the tannin extraction process at the industrial facility. The wood materials were air dried for several weeks and cut to pass through a 35 mesh sieve in an IKA M10.1 cutting mill (IKA ®‐Werke GmbH & Co. KG, Staufen, Germany). The final result was a 0.25 mm wood powder.

### Methods

#### Hydrothermal Reactor

The VW and DT char was obtained as a solid product of an hydrolysis conversion carried out in a Parr 4560 mini‐batch reactor following Mohan et al. methodology (78). H_2_SO_4_ 99 % used was from Sigma‐Aldrich, Milan, Italy.

#### Soxhlet Extraction with Acetone

The DT and VW's hydrochar were Soxhlet‐extracted for 6 hours with acetone (Sigma‐Aldrich, Milan, Italy) in a round bottomed flask. After extraction, the extract was concentrated with rotary evaporator (Rotavapor® Advanced R‐210 equipped with a Vacuum controller V‐850, Buchi, Flawil, Switzerland).

#### Mild Acidolysis

The DT and VW's hydrochar were cooked under reflux using a dioxane (Sigma‐Aldrich, Milan, Italy) ‐water (400 mL) in a volumetric ratio of 82 : 18 in a 0.1 M HCl solution for two hours. At the end, the solution was filtered for removal of the acidolysis residue (namely, *exhausted*). The filtration residue was washed with a portion of 200 mL dioxane‐water (82 : 18 vol%). In order to precipitate the dissolved lignin in the filtrate, dioxane was removed by rotary‐evaporation and replace with water of equal volume fractions. Finally, the solution was vacuum‐filtered and the collected lignin was further washed with acidified (pH 2) distilled water to remove sugar residues. Lignin was then put under the fume‐hood for 2 days and left to dry.

#### Calculation of Yield

The yield for each process step was calculated using the following equation:

$$yield\;\left(\%\right)=\frac{quantity\;obtained\;\left(g\right)}{initial\;quantity\;\left(g\right)}x\;100$$

In this equation the initial quantity refers to the mass of the material loaded in the process (25 g) and the quantity obtained refers to the mass of the material exiting the process.

### Fourier‐Transform Infrared Spectroscopy (FTIR)

Samples of char powder after the hydrothermal treatment, after the acetone extraction and after the mild acidolysis for both the char and the extracted lignin were analyzed through FTIR.

The char powders and the lignin were mixed with potassium bromide (KBr) to obtain pellets (Ø=7 mm) with a sample concentration of 2 wt % using a Specac mini‐pellets press at 2 bar for 5 minutes (Specac Inc., Orpington, UK). These FTIR spectra were recorder in absorbance mode in the range of 4000–400 cm^−1^ with a FTIR‐4100 Fourier Transform Infrared spectrometer (Jasco Corp., Easton, MD, USA).

Samples of the char after acetone extraction and of the exhausted char after the mild acidolysis were analyzed through FTIR in diffuse reflectance modality (DRIFT). A Nicolet Avatar 360 FTIR spectrometer was used in the range of 4000–400 cm^−1^. For each sample 1 mg was mixed with KBr that was also used as background reference.

All FTIR spectra were smoothed and baseline corrected. Although the infrared scanning was done in the region between 4000 and 400 cm^−1^, data evaluation was limited to the fingerprint region (1800–900 cm^−1^) in which most of the variations of infrared absorption occur.

### Scanning Electron Microscopy (SEM)

The char powders after the hydrothermal treatment, after the acetone extraction and after the mild acidolysis for both the char and the extracted lignin were attached to aluminum stubs using carbon tape and sputter‐coated with gold in a Balzers MED 010 unit. SEM analysis was conducted with a JSM 6010LA electron microscope (JEOL Limited, Tokyo, Japan). Size measurements were conducted on the SEM micrographs using the Adobe Photoshop CS4 extended software package (Adobe Systems, San Jose, CA, USA).

### Folin‐Ciocalteu Analysis

The char powders after the hydrothermal treatment, after the acetone extraction and after the mild acidolysis for both the char and the extracted lignin were analyzed through the Folin‐Ciocalteu method. An amount of 20 mg was solubilized in 20 mL of a methanol (Sigma‐Aldrich, Milan, Italy) – water 80 : 20 vol % solution for 3 hours at room temperature. 50 μL of such extraction solution were added to 1.2 mL of water and 1.25 mL of the FC reagent. After mixing, the samples were put at rest for 6 minutes, then 12.5 mL of a 7 % solution of NaCO_3_ were added and the samples were put at rest for 90 minutes at room temperature. Absorbance measurement at 760 nm were achieved by a Perkin‐Elmer Lambda 20 two‐rays spectrophotometer. Data analysis was performed by means of the linear regression of 7 gallic acid solutions absorbance data obtained as described above.

### High Performance Size Exclusion Chromatography (HPSEC)

The molecular weights of the lignin in the hydrochar after the mild acidolysis were determined by HP‐SEC. The analysis was performed using an Agilent 1100 HPLC system equipped with a temperated column compartment and a diode array detector using Agilent Chemstation (version B.03.02) for operation (Agilent Technologies Inc., Santa Clara, CA, USA). Molar weight analysis was performed using a PSS MCX column with 5 μm particle size and 1000 Å pore size (PSS Polymer Standard Services, Mainz, Germany) which was kept at 40 °C. The mobile phase was a 10 mM NaOH solution with addition of 20 mM NaNO_3_ as neutral salt. The flow rate was set to 0.5 mL/min and the UV signal at 280 nm was used for molar mass determination. For calibration, polystyrene sulfonate standards were used with the following molar masses at peak maximum: 65.400, 33.500, 15.800, 6430, 1670, 891 and 208 Da.

### Solubility Tests

5 mg of each samples were weighed and placed in 1 mL of DMSO 99.9 % (Sigma‐Aldrich, Milan, Italy) in separate glass vials. The mixtures were left at room temperature for 24 hours to ensure optimal interaction between the solvent and the sample. Then, the solutions were visually inspected for any solid residue.

### 2D HSQC NMR

2D heteronuclear single quantum coherence (HSQC) NMR spectra of the lignin_char samples were recorded on a Bruker AVANCE NEO 600 MHz spectrometer equipped with BBFO probe following the reported protocol.^[79]^ Approximately 80 mg of the sample was dissolved in 0.60 mL of DMSO‐d_6_, which was used as an internal reference point (*δ*_c_ 39.5; *δ*_h_ 2.50 ppm).

### Thermogravimetric Analysis (TGA)

A Thermogravimetric Analyzer TGA/DSC 3+ STARe System (Mettler Toledo, Columbus, OH, USA) was used to investigate the combustion properties. The samples were placed in a 150 μl Al_2_O_3_ pan and heated from 25 °C–800 °C at a heating rate of 40 °C/min under a nitrogen atmosphere. The samples were then cooled to 400 °C while maintaining the nitrogen atmosphere. Subsequently, the atmosphere was switched to oxygen, and the samples were reheated at 800 °C. A gas flow rate of 50 mL/min was used throughout the experiment.

## Conflict of Interests

The authors declare no conflict of interest.

1

## Data Availability

The data that support the findings of this study are available from the corresponding author upon reasonable request.
